# Supplementary material for: Efficacy and safety of etrolizumab in the treatment of inflammatory bowel disease: a meta-analysis
Source: PeerJ. 2024 Aug 23;12:e17945. doi: 10.7717/peerj.17945 (PMC11348897; doi:10.7717/peerj.17945)
Supplement: Supplemental Information 2 [file peerj-12-17945-s002.docx]

PubMed

(("etrolizumab" [Supplementary Concept]) OR (((((((etrolizumab[Title/Abstract]) OR (rhuMAb Beta7[Title/Abstract])) OR (ANTI-BETA7[Title/Abstract])) OR (ANTI-.BETA.7[Title/Abstract])) OR (RHUMAB .BETA.7[Title/Abstract])) OR (PRO145223[Title/Abstract])) OR (PRO-145223[Title/Abstract]))) AND (("Inflammatory Bowel Diseases"[Mesh]) OR ((((((((((((((((((((((((((Inflammatory Bowel Diseases[Title/Abstract]) OR (Inflammatory Bowel Disease[Title/Abstract])) OR (Bowel Diseases, Inflammatory[Title/Abstract])) OR (Colitis, Ulcerative[Title/Abstract])) OR (Idiopathic Proctocolitis[Title/Abstract])) OR (Ulcerative Colitis[Title/Abstract])) OR (Colitis Gravis[Title/Abstract])) OR (Inflammatory Bowel Disease, Ulcerative[Title/Abstract])) OR (Colitis Type[Title/Abstract])) OR (Crohn Disease[Title/Abstract])) OR (Crohn's Enteritis[Title/Abstract])) OR (Regional Enteritis[Title/Abstract])) OR (Crohn's Disease[Title/Abstract])) OR (Crohns Disease[Title/Abstract])) OR (Inflammatory Bowel Disease 1[Title/Abstract])) OR (Enteritis, Granulomatous[Title/Abstract])) OR (Granulomatous Enteritis[Title/Abstract])) OR (Enteritis, Regional[Title/Abstract])) OR (Ileocolitis[Title/Abstract])) OR (Colitis, Granulomatous[Title/Abstract])) OR (Granulomatous Colitis[Title/Abstract])) OR (Ileitis, Terminal[Title/Abstract])) OR (Terminal Ileitis[Title/Abstract])) OR (Ileitis, Regional[Title/Abstract])) OR (Regional Ileitides[Title/Abstract])) OR (Regional Ileitis[Title/Abstract])))

Embase

| No. | Query |
| --- | --- |
| #36 | #8 AND #35 |
| #35 | #9 OR #10 OR #11 OR #12 OR #13 OR #14 OR #15 OR #16 OR #17 OR #18 OR #19 OR #20 OR #21 OR #22 OR #23 OR #24 OR #25 OR #26 OR #27 OR #28 OR #29 OR #30 OR #31 OR #32 OR #33 OR #34 |
| #34 | 'regional ileitis':ab,ti |
| #33 | 'regional ileitides':ab,ti |
| #32 | 'ileitis, regional':ab,ti |
| #31 | 'terminal ileitis':ab,ti |
| #30 | 'ileitis, terminal':ab,ti |
| #29 | 'granulomatous colitis':ab,ti |
| #28 | 'colitis, granulomatous':ab,ti |
| #27 | 'ileocolitis':ab,ti |
| #26 | 'enteritis, regional':ab,ti |
| #25 | 'granulomatous enteritis':ab,ti |
| #24 | 'enteritis, granulomatous':ab,ti |
| #23 | 'inflammatory bowel disease 1':ab,ti |
| #22 | 'crohns disease':ab,ti |
| #21 | 'crohns disease':ab,ti |
| #20 | 'regional enteritis':ab,ti |
| #19 | 'crohns enteritis':ab,ti |
| #18 | 'crohn disease':ab,ti |
| #17 | 'colitis type':ab,ti |
| #16 | 'inflammatory bowel disease, ulcerative':ab,ti |
| #15 | 'colitis gravis':ab,ti |
| #14 | 'ulcerative colitis':ab,ti |
| #13 | 'idiopathic proctocolitis':ab,ti |
| #12 | 'colitis, ulcerative':ab,ti |
| #11 | 'bowel diseases, inflammatory':ab,ti |
| #10 | 'inflammatory bowel disease':ab,ti |
| #9 | 'inflammatory bowel disease'/exp |
| #8 | #1 OR #2 OR #3 OR #4 OR #5 OR #6 OR #7 |
| #7 | 'pro-145223':ab,ti |
| #6 | 'pro145223':ab,ti |
| #5 | 'rhumab .beta.7':ab,ti |
| #4 | 'anti-.beta.7':ab,ti |
| #3 | 'anti-beta7':ab,ti |
| #2 | 'rhumab beta7':ab,ti |
| #1 | 'etrolizumab'/exp |

Cochrane

ID Search Hits

#1 MeSH descriptor: [] explode all trees 0

#2 (rhuMAb Beta7):ti,ab,kw OR (ANTI-BETA7):ti,ab,kw OR (ANTI-.BETA.7):ti,ab,kw OR (RHUMAB .BETA.7):ti,ab,kw OR (PRO145223):ti,ab,kw 8

#3 (PRO-145223):ti,ab,kw 0

#4 #1 or #2 or #3 8

#5 MeSH descriptor: [Inflammatory Bowel Diseases] explode all trees 4897

#6 (Inflammatory Bowel Disease):ti,ab,kw OR (Bowel Diseases, Inflammatory):ti,ab,kw OR (Colitis, Ulcerative):ti,ab,kw OR (Idiopathic Proctocolitis):ti,ab,kw OR (Ulcerative Colitis):ti,ab,kw 9276

#7 (Colitis Gravis):ti,ab,kw OR (Inflammatory Bowel Disease, Ulcerative):ti,ab,kw OR. (Colitis Type):ti,ab,kw OR (Crohn Disease):ti,ab,kw OR (Crohn's Enteritis):ti,ab,kw 6874

#8 (Regional Enteritis):ti,ab,kw OR (Crohn's Disease):ti,ab,kw OR (Crohns. Disease):ti,ab,kw OR (Inflammatory Bowel Disease 1):ti,ab,kw OR (Enteritis, Granulomatous):ti,ab,kw 7629

#9 (Granulomatous Enteritis):ti,ab,kw OR (Enteritis, Regional):ti,ab,kw OR. (Ileocolitis):ti,ab,kw OR (Colitis, Granulomatous):ti,ab,kw OR (Granulomatous Colitis):ti,ab,kw 119

#10 (Ileitis, Terminal):ti,ab,kw OR (Terminal Ileitis):ti,ab,kw OR (Ileitis, Regional):ti,ab,kw NOT (Regional Ileitides):ti,ab,kw OR (Regional Ileitis):ti,ab,kw 31

#11 #5 or #6 or #7 or #8 or #9 or #10 12851

#12 #4 and #11 8

web of science

| # |  |
| --- | --- |
| 1 | TS=(etrolizumab) OR TS=(rhuMAb Beta7) OR TS=(ANTI-BETA7) OR TS=(ANTI-.BETA.7) OR TS=(RHUMAB .BETA.7)OR TS=(PRO145223) OR TS=(PRO-145223) |
| 2 | TS=(Inflammatory Bowel Diseases) OR TS=(Inflammatory Bowel Disease) OR TS=(Bowel Diseases, Inflammatory ) OR TS=(Colitis, Ulcerative) OR TS=(Idiopathic Proctocolitis) OR TS=(Ulcerative Colitis) OR TS=(Colitis Gravis) OR TS=(Inflammatory Bowel Disease, Ulcerative ) OR TS=(Colitis Type) OR TS=(Crohn Disease) OR TS=(Crohn's Enteritis) OR TS=(Regional Enteritis) OR TS=(Crohn's Disease) OR TS=(Crohns Disease) OR TS=(Inflammatory Bowel Disease 1) OR TS=(Enteritis, Granulomatous) OR TS=(Granulomatous Enteritis) OR TS=(Enteritis, Regional) OR TS=(Ileocolitis) OR TS=(Colitis, Granulomatous) OR TS=(Granulomatous Colitis) OR TS=(Ileitis, Terminal) OR TS=(Terminal Ileitis) OR TS=(Ileitis, Regional) OR TS=(Regional Ileitides) OR TS=(Regional Ileitis) |
| 3 | #1 AND #2 |
