# Supplementary material for: Efficacy and safety of etrolizumab in the treatment of inflammatory bowel disease: a meta-analysis
Source: PeerJ. 2024 Aug 23;12:e17945. doi: 10.7717/peerj.17945 (PMC11348897; doi:10.7717/peerj.17945)

Table s1 Grade of evidence

| Outcome | Grade |
| --- | --- |
| Clinical Remission | Moderate |
| Clinical Response | Moderate |
| Endoscopic Remission | Moderate |
| endoscopic improvement | Low |
| Histological remission | Low |
| adverse events | Moderate |

figure S1 Risk of bias graph


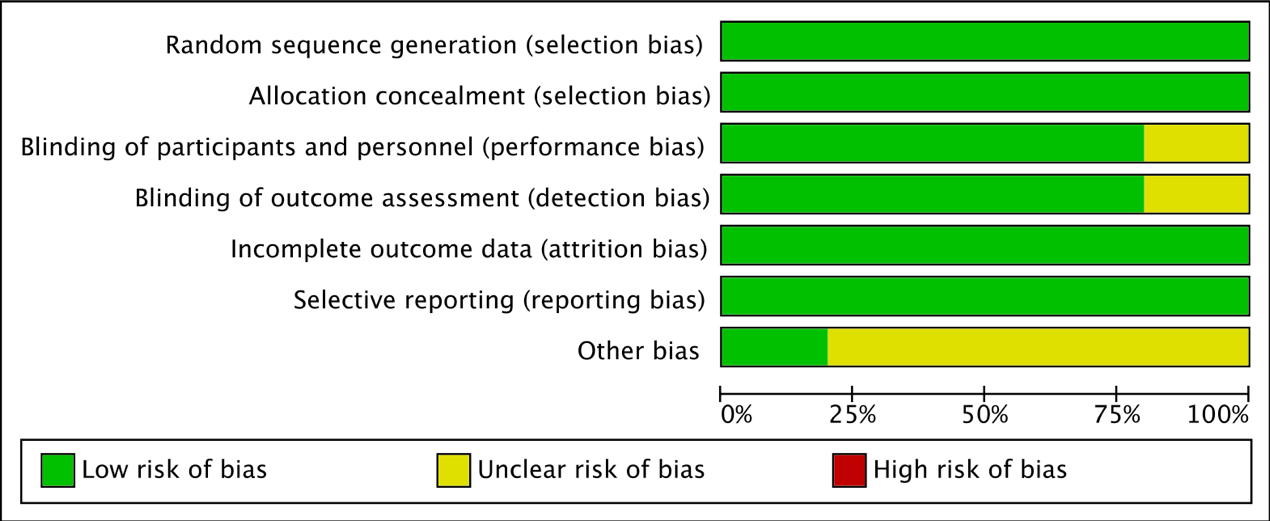


figure S2 Risk of bias summary


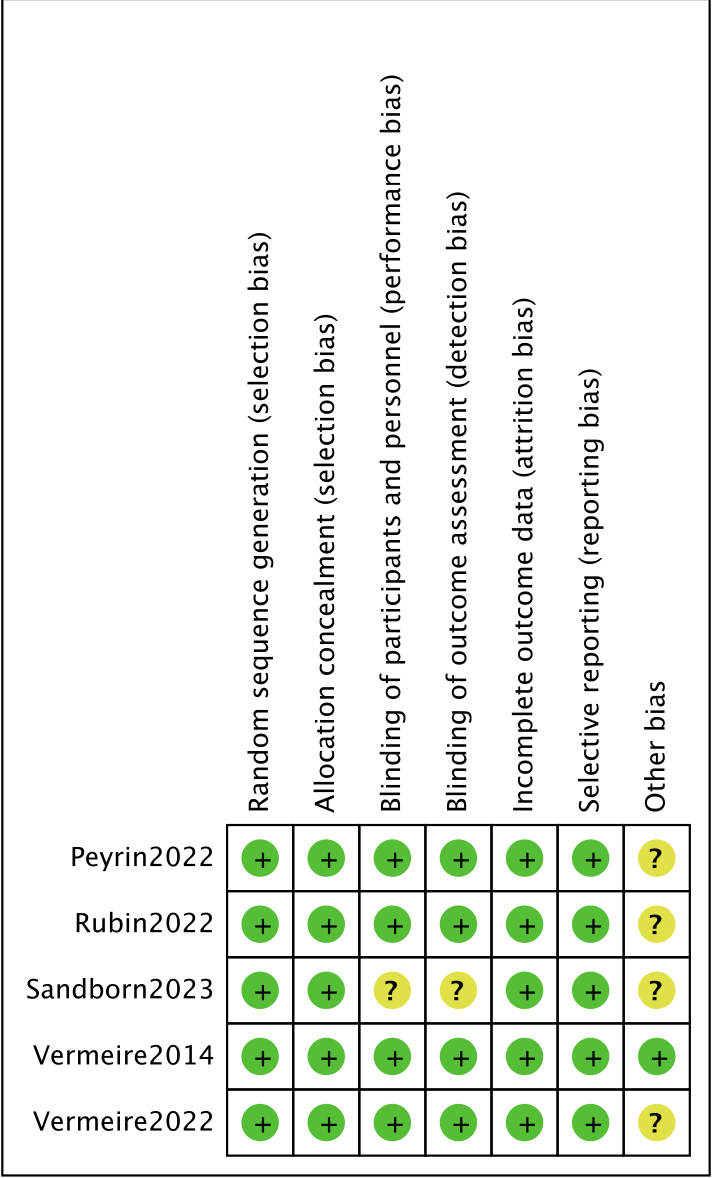


figure s3 egger test of clinical remission


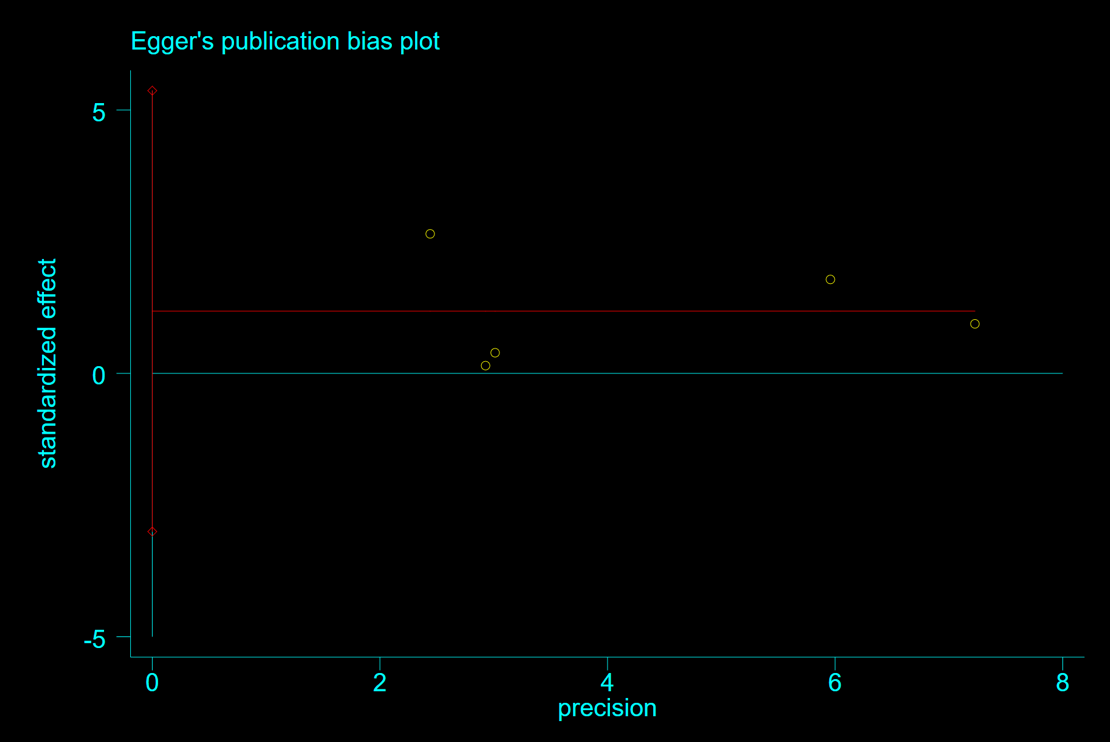


figure s4 egger test of endoscopic improvement


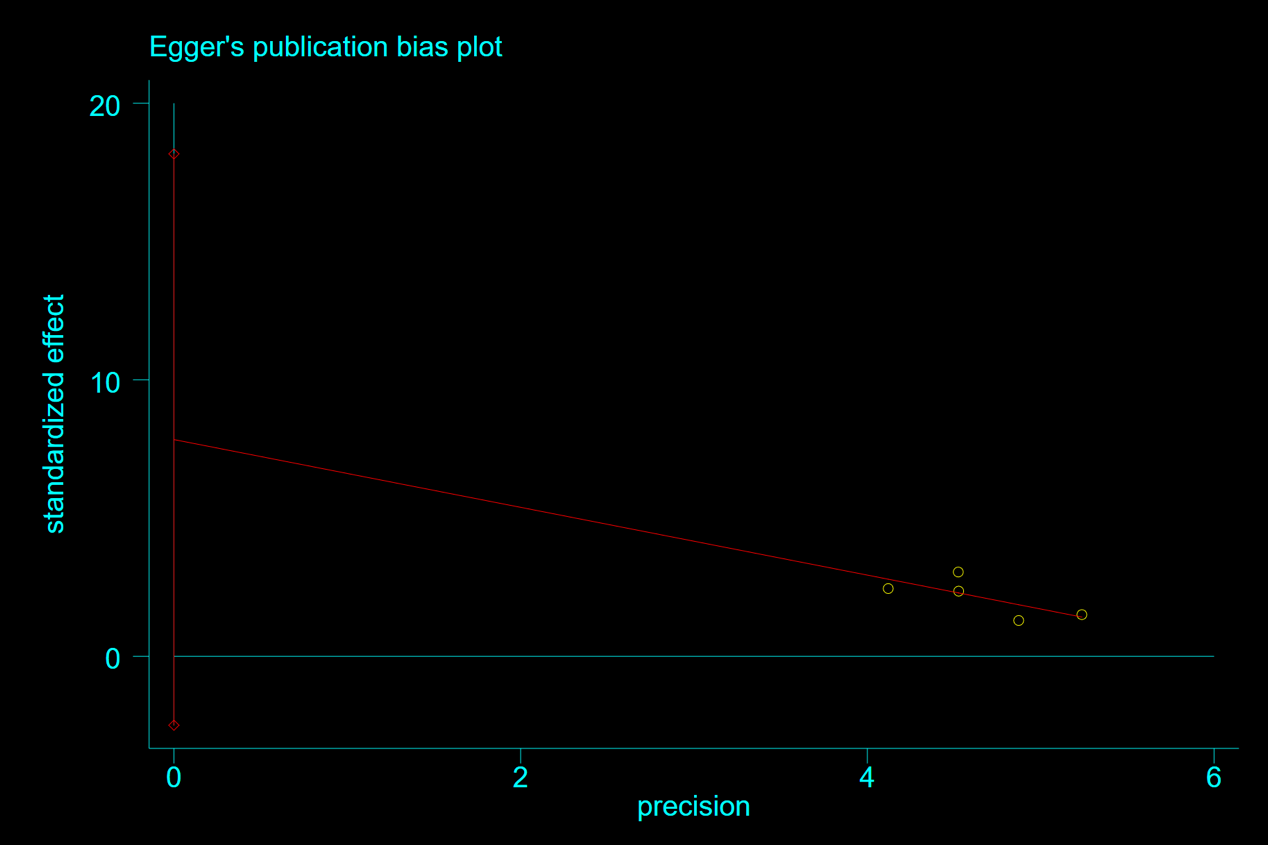


figure s5 egger test of adverse events


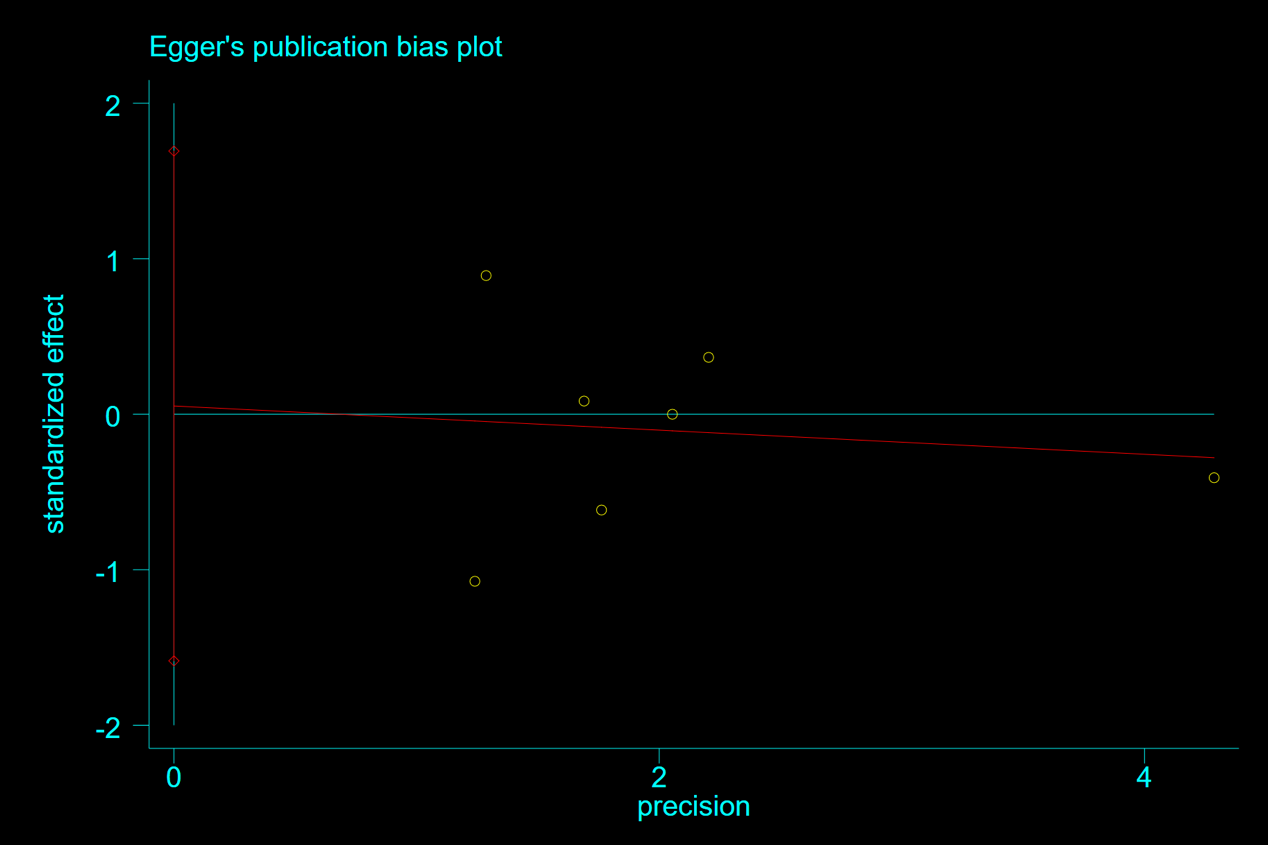


figure s6 egger test of histological remission


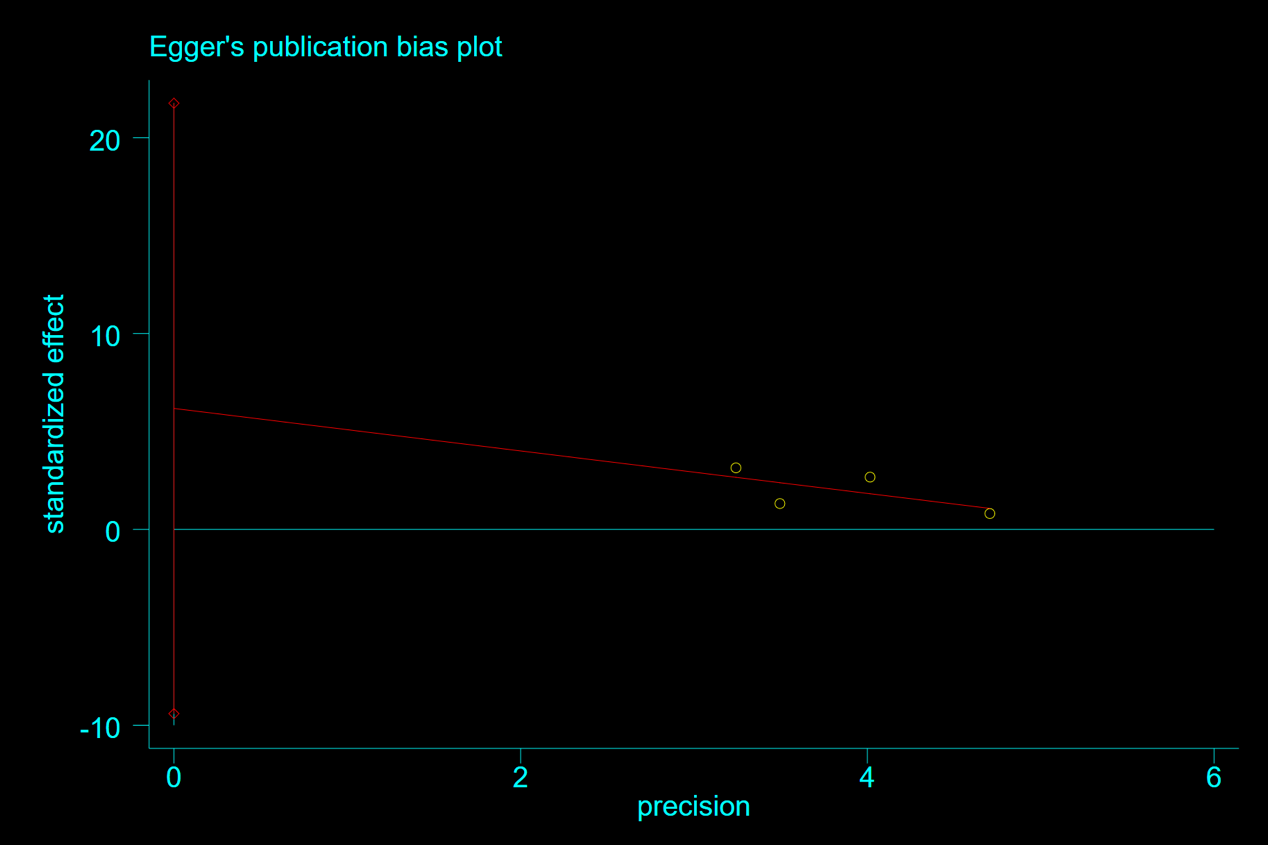


figure s7 egger test of clinical response


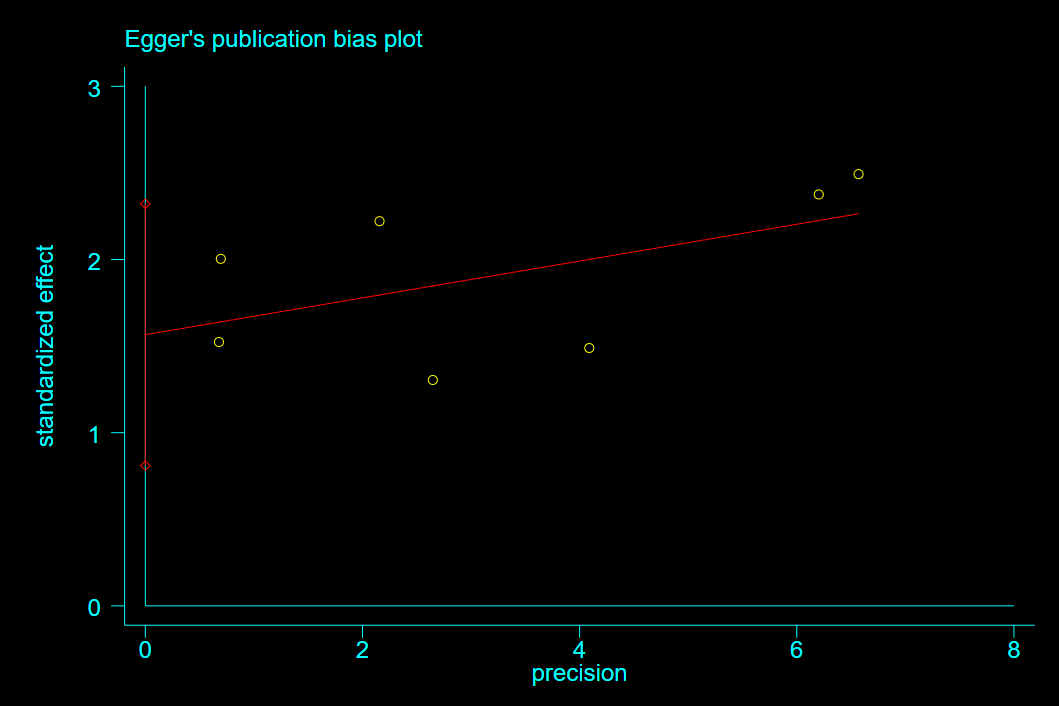


figure s8 egger test of clinical remission


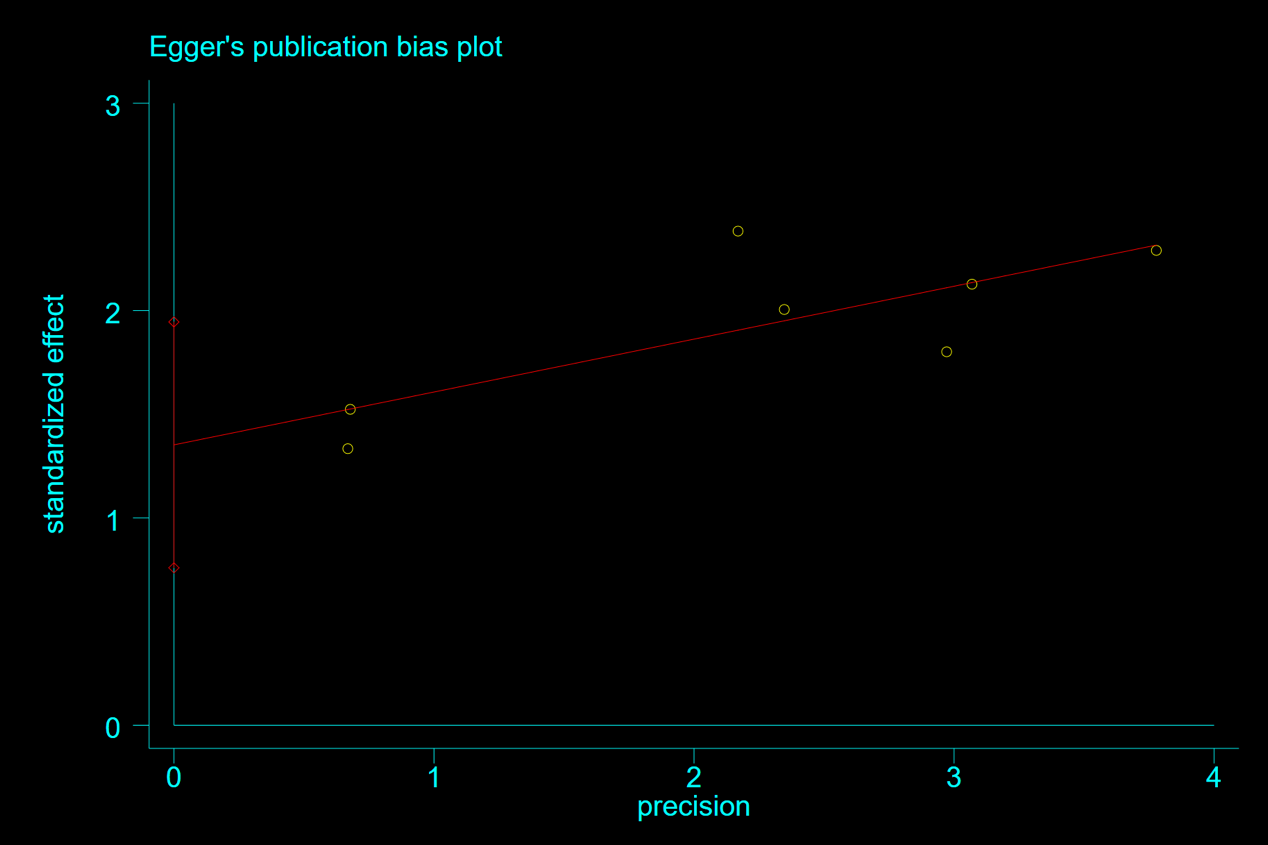

Supplement: Supplemental Information 3 [file peerj-12-17945-s003.docx]
