# Supplementary material for: Efficacy and safety of etrolizumab in the treatment of inflammatory bowel disease: a meta-analysis
Source: PeerJ. 2024 Aug 23;12:e17945. doi: 10.7717/peerj.17945 (PMC11348897; doi:10.7717/peerj.17945)
Supplement: Supplemental Information 4 [file peerj-12-17945-s004.docx]

1. The rationale for conducting the systematic review / meta-analysis;

responds: Etrolizumab is a humanized monoclonal IgG1 antibody directed against the β7 subunit of the heterodimeric integrins α4β7 and αEβ7. α4β7 integrin is a key mediator of leukocyte infiltration in the gastrointestinal tract by interacting with MAdCAM-1 on the vascular endothelium of mucosal tissues. However, the efficacy and safety of etrolizumab for IBD are still controversial, so we hope to resolve these controversies with this study and provide new options for clinical patient treatment.

1. The contribution that it makes to knowledge in light of previously published related reports, including other meta-analyses and systematic reviews.

responds: As far as we are concerned, this is not the first time etrolizumab treatment for IBD has been evaluated, but a previous Cochrane study(27) included only two original studies. a 2019 meta-analysis(28) compared an indirect comparison of etrolizumab and infliximab. This is not consistent with our current meta-analysis inclusion metrics. Therefore, the current study included more high-quality studies and the conclusions are more credible. Our study confirms that etrolizumab is a promising drug in IBD. This suggests that etrolizumab can be used to improve response in future IBD patients.
